# Glycogen Synthase Kinase-3 Inhibition Enhances Translation of Pluripotency-Associated Transcription Factors to Contribute to Maintenance of Mouse Embryonic Stem Cell Self-Renewal

**DOI:** 10.1371/journal.pone.0060148

**Published:** 2013-04-05

**Authors:** Yolanda Sanchez-Ripoll, Heather K. Bone, Tom Owen, Ana M. V. Guedes, Elsa Abranches, Benjamin Kumpfmueller, Ruth V. Spriggs, Domingos Henrique, Melanie J. Welham

**Affiliations:** 1 Centre for Regenerative Medicine and Department of Pharmacy & Pharmacology, University of Bath, Bath, United Kingdom; 2 Medical Research Centre Toxicology Unit, University of Leicester, Hodgkin Building, Leicester, United Kingdom; 3 Instituto Medicina Molecular, and Instituto de Histologia e Biologia do Desenvolvimento, Faculdade de Medicina da Universidade de Lisboa, Lisbon, Portugal; Baylor College of Medicine, United States of America

## Abstract

Maintenance of embryonic stem cell (ESC) self-renewal and pluripotency are controlled by extrinsic factors, molecular signaling pathways and transcriptional regulators. While many of the key players have been studied in depth, how the molecular signals interact with transcription factors of the pluripotency network to regulate their action remains less well understood. Inhibition of glycogen synthase kinase 3 (Gsk-3) has been implicated in the maintenance of mouse ESC pluripotency, although there is contradictory data on its role, with enhancement of cell survival and metabolism, stabilisation of c-Myc and activation of Wnt signalling proposed as potential mechanisms. We have discovered that suppression of Gsk-3 activity leads to enhanced protein levels of key transcriptional regulators of the pluripotency network, notably Nanog, Tbx3 and c-Myc. Protein stability was unchanged following Gsk-3 inhibition, although interestingly, Nanog and Tbx3 proteins were found to have half-lives of 1–3 h, while that of Oct4 protein was longer, at 6 h. We demonstrate that the effects on protein levels seen following inhibition of Gsk-3 are due to both enhanced *de novo* synthesis of Nanog protein and increases in the proportion of Nanog and Tbx3 RNAs bound to polysomes, findings consistent with Gsk-3 regulating translation of these factors. These effects were not due to changes in regulators of general translation initiation machinery nor mediated via the 5′ or 3′ UTR sequences of Nanog alone. The data we present provide both new conceptual insight into the mechanisms regulated by Gsk-3 that may contribute to ESC self-renewal and, importantly, establish control of protein translation as an additional mechanism involved in modulation of ESC pluripotency.

## Introduction

ESC pluripotency is regulated by the coordinated action of extrinsic factors, signaling pathways and an intrinsic network of transcription factors [1], [2]. Leukemia Inhibitory Factor (LIF) is a key factor for maintenance of mouse ESC self-renewal [3], its actions mediated via Stat3 signalling [4], [5], [6] and c-Myc [7]. LIF also activates the extracellular regulated kinases Erk1 and Erk2, which promote differentiation [8], Src kinases [9], [10], Ribosomal S6 kinases [10], and Phosphoinositide 3-Kinase (PI3K) signaling [11]. Serum or Bone Morphogenetic Proteins 2 or 4 (BMP 2/4) are also required and cooperate with LIF to maintain self-renewal [12]. Several studies have demonstrated that inhibition of Glycogen synthase kinase 3 (Gsk-3) enhances self-renewal of mouse ESCs [13], [14], [15] and can sustain self-renewal of ESCs grown on mouse embryo fibroblast feeders in the absence of LIF [16], while mouse ESCs in which both Gsk-3 isoforms (α and ß) have been deleted (DKO ESC [17]) are more resistant to differentiation. Indeed, mouse ESC pluripotency can be maintained in serum-free media in the absence of LIF and BMP4 by simultaneous inhibition of Gsk-3 and Mitogen activated and extracellular-regulated kinase kinase (MEK), referred to as ‘2i conditions’ or the ground state of pluripotency [15].

The transcription factors Oct4, Sox2 and Nanog have been termed ‘master regulators’ owing to the important role they play in specifying and maintaining ESC pluripotency [1], [2]. Nanog is essential for establishment of pluripotency in the inner cell mass [18] and although it is not absolutely required for maintenance of ESC self-renewal [19] over-expression of Nanog can maintain ESC self-renewal in the absence of LIF [18], [20]. Levels of Oct4 are key to maintaining pluripotency [21] and the finding that Oct4, Sox2 and Nanog bind to many of the same promoter sequences has led to the proposal that they form a regulatory network which reinforces pluripotency [22], [23]. Other transcription factors also contribute to maintenance of the ESC state and include Tbx3, which is regulated by LIF and PI3K-dependent pathways [24], [25] and c-Myc, ectopic expression of which can relieve the need for LIF/STAT3 signaling [7]. The nuclear receptor Esrrb can also sustain self-renewal of ESCs and has recently been shown to be a key transcriptional target of Nanog [26], as well as being downstream of Gsk-3 and Tcf3 [27]. Despite this knowledge, we still lack a detailed understanding of how the molecular signals implicated in control of self-renewal interact with the intrinsic network of pluripotency-associated transcription factors, in large part due to the focus on transcriptional regulation.

The dynamic transcriptional control of Nanog and other ESC-expressed factors such as Rex1 and Esrrb has been reported [19], [28], implying that in a pluripotent state ESCs are primed to respond to environmental signals, whether that signal promotes pluripotency or differentiation. However, transcriptional changes can be slow in comparison to post-transcriptional mechanisms so, conceptually, regulation of protein, rather than RNA levels would endow ESCs with the ability to respond rapidly to changes in the environment. Intriguingly, very little is currently known about the dynamics of pluripotency transcription factor protein expression and the regulatory mechanisms involved. Here, we demonstrate that a key regulator of mouse ESC pluripotency, Gsk-3, controls the protein levels of key members of the pluripotency network of transcription factors by post-transcriptional mechanisms. Acute inhibition of Gsk-3 led to up-regulation of protein expression of Nanog and Tbx3. At early stages following Gsk-3 inhibition enhanced protein synthesis was observed, which preceded increases in transcription. Furthermore, inhibition of Gsk-3 increased the proportion of Nanog and Tbx3 transcripts associated with polyribosomes, consistent with enhanced translation. By demonstrating that control of protein translation by Gsk-3-dependent signaling regulates levels of key transcription factors, our results provide new conceptual insight into the mechanisms contributing to ESC pluripotency.

## Materials and Methods

### Embryonic Stem Cell Culture

The mESC lines, E14tg2a [29], Nanog-TNG [19], Gsk-3 DKO and Gsk-3 WT [17] were cultured on tissue culture dishes coated with 0.1% (w/v) gelatin with Knock-Out (KO) Dulbecco’s modified Eagle medium (Invitrogen) in the presence of 1000 units/ml murine LIF (ESGRO; Chemicon) as previously described [11]. The Nanog reporter mESC line, referred to as Nd-ESC, was derived from E14tg2a ES cells and contains a BAC transgene in which the short-lived fluorescent protein VNP (Venus) has been placed under the control of Nanog regulatory regions. The generation and characterisation of this cell line are described in detail elsewhere [30]. The Nd-ESC reporter line was routinely grown in Glasgow Modified Eagle Medium (GMEM, Invitrogen), supplemented with 10% (v/v) fetal bovine serum (FBS) (ES-qualified, Invitrogen) and 2 ng/ml LIF. For time-course analyses with Gsk-3 inhibitors, ESCs were cultured in GMEM supplemented with 10% (v/v) ES-tested Fetal Bovine Serum (FBS) (Hyclone, ThermoFisher) and 1000 U/ml LIF or in defined N2B27 medium supplemented with 1000 U/ml LIF (Chemicon) and 10 ng/ml BMP4 (Stemgent). Defined N2B27 medium consists of 1 volume DMEM F-12 medium: 1 volume Neurobasal medium (Invitrogen) supplemented with N2 and B27 supplements (Invitrogen), 0.0125% (v/v) Monothioglycerol (Sigma), 50 mg/ml bovine serum albumin (BSA) (Sigma), 2 mM Glutamine (Invitrogen), as previously described [12], [31]. Gsk-3 inhibitors used were CHIR 99201 (Axon MedChem) and 1 m [14]. mESCs were also cultured in N2B27 in the absence of LIF and BMP4 and the presence of 3 µM CHIR99201 and 1 µM PD0325901 (MEK inhibitor; Axon MedChem) ‘2i conditions’, as previously described [15].

### Measurement of ESC Growth

Cells were plated at 2×10^5^ cells/5 cm dish in GMEM supplemented with FBS and LIF, N2B27 supplemented with LIF and BMP4 or N2B27 with DMSO (Control), 3 µM Gsk-3 inhibitor (CHIR) or 1 µM MEK inhibitor (PD0325901) for 3 days. Dishes were counted in triplicate using Trypan Blue dye.

### Time-lapse Confocal Microscopy, Flow Cytometry and Cell Sorting

For time-lapse confocal microscopy, 24 h after plating in serum plus LIF Nanog-TNG mESCs [19] were equilibrated for 1 h at 37°C on the confocal microscope prior to addition of 3 µM CHIR99201, or DMSO as a control. Cell populations were imaged by time-lapse for 24 hours, with pictures taken every 10 minutes. 10 random fields, each containing approximately 15 colonies, were recorded for each condition. Fluorescence-activated cell sorting, performed with a FACS Aria instrument (Becton Dickinson) was used to sort Nanog:VNP-low populations of Nd-ESCs. Live cells were gated based on forward and side scatter. Immediately after sorting, cell viability was determined and Nanog:VNP-low cells were plated at 5×10^4^ cells/cm^2^ onto gelatin-coated dishes in the presence of serum and LIF, and incubated in the presence of 3 µM CHIR99201 or DMSO (as a control). Cells were followed for 24 h, during which cell morphology and the percentage of Nanog:VNP positive cells were analysed (time points –0, 4, 8, 12 and 24 h). E14tg2a cells were used as a negative control, to obtain the positive gate region. Nanog:VNP analyses were performed on a FACS Calibur flow cytometer (Becton Dickinson).

### Assessment of Protein Stability and Re-synthesis

To assess protein stability, ESCs were cultured in GMEM supplemented with LIF and serum or N2B27 supplemented with BMP4 and LIF overnight before adding 10 µg/ml of cycloheximide. Protein extracts were obtained after 0, 1, 3 and 6 hours of cycloheximide addition using the NE-PER Nuclear and Cytoplasmic Extraction Reagents (Pierce) following the Manufacturer’s instructions. Protein concentrations were measured using the Bio-Rad protein assay reagent following the Manufacturer’s instructions. To investigate protein re-synthesis, cells were cultured as above, treated with 10 µg/ml cycloheximide for 4 hours after which cells were washed extensively before addition of fresh medium free of cycloheximide. Protein extracts were obtained using the NE-PER nuclear and cytoplasmic extraction reagent (Pierce).

### SDS-PAGE and Immunoblotting

Cell lysates were separated by SDS-PAGE using 10 and 12% polyacrylamide gels and transferred to nitrocellulose membranes by immunoblotting in semi-dry transfer buffer as described previously [32]. Blots were probed with the following antibodies using the concentrations stated: 1∶2000 anti-Oct4 (Santa Cruz Biotechnology; sc-9801), 1∶1000 anti-Nanog (Abcam; Ab80892); 1∶1000 anti-c-Myc (Cell signalling, 9402), 1∶750 anti-Tbx3 (Santa Cruz Biotechnology; sc17871) and 1∶15000 anti-Gapdh (Ambion; AM4300). Goat anti-rabbit, rabbit anti-goat or goat anti-mouse antibodies conjugated to horseradish peroxidase (DAKO) were used at 1∶20,000 and blots were developed using Chemiglow (GRI/AlphaInnotech) or ECL Prime (GE Healthcare) according to the manufacturer’s directions. Blots were stripped and reprobed as previously described [32].

### Polysomal Fractionation and RNA Isolation

ESCs grown in GMEM supplemented with LIF and serum were treated as required and polysomal fractionation carried out by differential sucrose density centrifugation as described in [33], with minor amendments. Briefly, prior to cell lysis, 150 µg/ml cycloheximide was added for 15 minutes to stop ribosome movement. Cells were then lysed using buffer described previously [32] supplemented with 2 mM DTT, 150 µg/ml cycloheximide and 80 U/ml RNAsin. Cell lysates were centrifuged for 3 minutes at 4°C and 6000 rpm to remove the nuclei and the supernatant transferred to new tubes and re-centrifuged at 4°C and 16,000 rpm for 5–10 minutes to remove mitochondria and membrane particles. Supernatant from equivalent cell numbers for each sample was loaded into a sucrose gradient column made in DEPC-treated water with 10 mM Tris-Hcl pH 7.5, 140 mM NaCl, 1.5 mM MgCl_2_ and 10% (w/v), 15% (w/v), 20% (w/v), 35% (w/v), 40% (w/v) and 50% (w/v) sucrose, 1 mM DTT and 100 µM cycloheximide. Sedimentation was performed by centrifugation at 130,000×g for 1.5 hr at 4°C in an SW40-Ti swinging bucket rotor (Beckman) [33]. After centrifugation, fractions were collected from the top of the gradient and transferred to tubes containing 0.5% (w/v) SDS, 12 µl EDTA (stock 0.5 M) and 10 µl proteinase K (stock 100 mg/ml), mixed immediately and incubated at 37°C for 30 minutes. After incubation, 200 ng yeast tRNA was added to assist with precipitation. Samples were extracted using phenol:chloroform (1∶1) and RNA precipitated from the aqueous phase. Washed and dried RNA pellets were resuspended in 20 µl DEPC-treated water. OD at 260 nm was measured to determine polysomal distribution and equal volumes of individual fractions were either pooled to generate monosomal (fractions 1–7) and polysomal (fractions 8–16) samples or analysed as individual fractions. LiCl precipitation was performed on the pooled fractions.

### Preparation of Total RNA

Total RNA was extracted using Trizol reagent (Invitrogen) following the manufactureŕs instructions. To remove contaminating genomic DNA, RNA samples were treated with RQ1 DNase. 1 µg of RNA was incubated with 1 U DNase (Promega) in DNase buffer (400 mM Tris-HCl pH8.0, 100 mM MgSO_4_, 10 mM CaCl_2_) (Promega) at 37°C for 30 minutes. DNase was heat inactivated at 65°C for 10 minutes with 1 µl DNase stop solution (20 mM EDTA pH8.0). Reverse transcription was carried out as previously described [34].

### Quantitative RT-PCR

qRT-PCR was performed using the LightCycler™ 1.5 system (Roche) as previously described [34]. Gene specific amplification was verified by performing melt-curve analysis after 40 amplification cycles. PCR efficiencies of both the target and the reference genes were calculated and the relative values for calibrator-normalised target gene expression determined using Lightcycler software (v4.0). Primer sequences used are shown in Table S1. Transcript levels were normalised to ß-actin and either a 2-way ANOVA followed by Bonferroni post-hoc test or two-tailed Student´s t-test were used to check statistical significance between samples.

### Bioinformatic Analysis of Nanog 5′ and 3′ UTR Sequences

Potential IRES sequences were identified by searching for polypyrimidine tracts (≥10) and then analysing for sequence motifs e.g. CCTCC, often found within such tracts in functional IRES. Potential upstream open reading frames (uORFs) were identified by looking for a start codon (AUG, or alternatives) in the 5′ UTR, an in-frame stop codon before the end of the coding sequence and a length of at least 9 nucleotides. All analyses were performed using in-house Perl scripts. AU-rich elements (ARE sites), that bind proteins such as TTP, HuR, and Auf1 to stabilise/destabilise mRNA, were searched for using AREsite [http://rna.tbi.univie.ac.at/cgi-bin/AREsite.cgi].

### Cloning of Nanog 5′ and 3′ UTR Sequences and Luciferase Reporter Assays

The 215bp 5′ UTR region of mouse Nanog gene (RefSeq NM_028016), was amplified with the following primers (Nanog 5′UTR forward primer sequence with NheI restriction site underlined - 5′ AATAGCTAGCTCTATCGCCTTGAGCCGTTGG 3′ and Nanog 5′UTR reverse primer 2 sequence with Xhol1 restriction site underlined - 5′ ATAAACTCGAGGTCAGTGTGATGGCGAGGGAA 3′) and cloned into NheI/XhoI restricted pGL3’ [35], [36] to generate pGL3’-Nanog-5′UTR. 222bp (R1) or 1048bp (R2) segments of the 3′ UTR region of mouse Nanog gene (RefSeq NM_028016), were amplified with the following primers (Nanog 3′UTR forward primer sequence with SpeI site underlined –5′ ATAATTACTAGTGACTTACGCAACATCTGGGCT 3′ and Nanog 5′ UTR reverse primer 1 5′ TTATAGAATTCCGACTGCTCTTCCGAAGGTC 3′ to generate 222 bp segment (R1) or reverse primer 2, to generate 1048 bp segment (R2), with EcoR1 site underlined) and cloned into SpeI/EcoRI restricted pGL3-MCS [35], [36] to generate pGL3-Nanog-3′UTR-R1 and R2 constructs. All constructs were verified by DNA sequencing. Mouse E14 ESCs were transiently transfected with 2 µg of either parental vector (pGL3’ or pGL3-MCS) or test vector (pGL3’-Nanog-5′UTR, pGL3-Nanog-3′UTR R1 or R2) along with 0.04 µg of Renilla plasmid using Lipofectamine 2000 as previously described [14]. 24 h after transfection cells were treated with DMSO (vehicle control) or Gsk-3 inhibitor 1 m at 2 µM and after a further 4–16 h cell extracts were prepared and dual luciferase assays performed as previously described [14].

## Results

### Gsk-3 Inhibition Regulates Expression of Pluripotency-associated Transcription Factor Proteins

**Figure 1:**
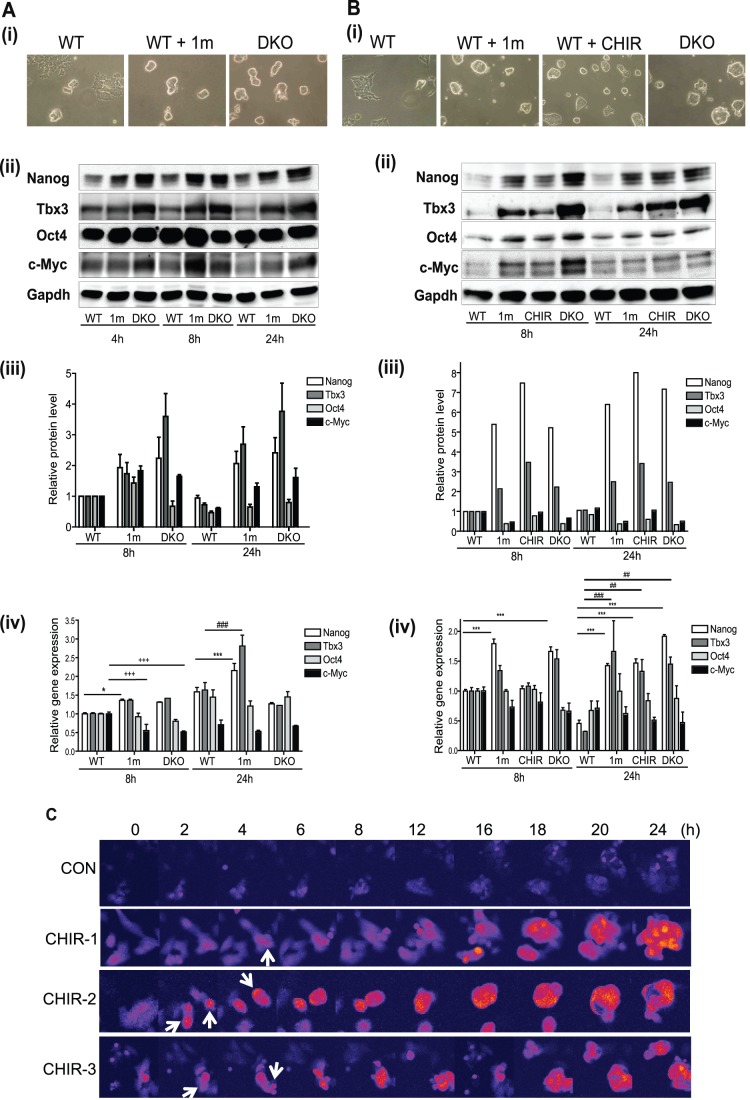
Inhibition or ablation of Gsk-3 enhances expression of Nanog, Tbx3 and c-Myc proteins in ESCs. E14tg2a wild-type (WT) and Gsk-3α/ß double knockout (DKO) mESCs were cultured in the presence of serum plus LIF (**A**) or chemically defined medium (N2B27) plus LIF and BMP4 (**B**). Gsk-3 inhibitors 1 m or CHIR99201 (CHIR) were added to WT ESCs at 2 and 3 µM respectively, as indicated in both **A** and **B**. (i) Images show colonies formed from WT ESCs that were untreated (WT), or cultured in the presence of 2 µM 1 m or 3 µM CHIR99201 for 48 h and Gsk-3 DKO ESCs. Protein and RNA were extracted at the times indicated. (ii) 12 µg of nuclear protein extracts were immunoblotted with the antibodies specified. (iii) Antibody signals were quantified and normalised to Gapdh. A value of one was given to normalised protein levels in WT ESCs at 8 hours and values for other samples related to these. The data show relative protein levels and are the average and SD of duplicate experiments for **A** and a single representative experiment in **B**. (iv) Quantitative RT-PCR was carried out and gene expression normalized relative to ß-actin levels. A value of one was given to normalised RNA levels in WT ESCs at 8 hr and other samples related to these. The data show relative gene expression levels and are the average and S.E.M of quadruplicate samples. *, p<0.05; **, p<0.01, ***, p<0.005. * for Nanog, #for Tbx3 and+for c-Myc (2-way ANOVA with Bonferroni post-hoc test). (**C**) Nanog-TNG reporter ESCs were cultured in serum and LIF in the presence of 3 µM CHIR99201 (CHIR) or DMSO (CON) and imaged over a period of 24 h using confocal microscopy, with images taken every 10 mins. Representative still images of ESC colonies are shown for the times specified. 10 fields of view chosen at random and within each field at least 15 colonies were imaged. White arrows indicate cells where Nanog reporter expression increases at early time points. Complete time-lapse series for control and CHIR-2 are available Videos S1 and S2.

Based on the range of roles reported for Gsk-3 in regulation of mouse ESC self-renewal and pluripotency [14], [15], [16], [27], [37], [38], [39], [40], [41], [42] and the gap in our understanding of how signaling pathways interact with the intrinsic network of pluripotency transcription factors, we decided to examine the impact of inhibition of Gsk-3 on protein levels of key regulators of ESC pluripotency. Protein and RNA levels for the transcription factors Nanog, Tbx3, Oct4 and c-Myc were measured in wild-type E14 ESCs cultured with Gsk-3 inhibitors (CHIR99201 [15] or 1 m [14]) and in ESCs derived from this line in which all four alleles of Gsk-3 have been knocked out ([17], Gsk-3 DKO ESCs). Previous studies have used various culture conditions to maintain mouse ESCs, commonly DMEM supplemented with serum and LIF or serum-free N2B27 supplemented with LIF and BMP4, so we felt it was important to analyse the effects of Gsk-3 inhibition under these different conditions. As shown in Fig 1A(i) and B(i) Gsk-3-inhibitor-treated WT ESCs and Gsk-3 DKO exhibited compact colony morphologies. Increased levels of Nanog and Tbx3 proteins (2-6-fold) were observed 4–8 hours after Gsk-3 inhibition was initiated in the presence of serum (Fig 1A (ii) & (iii)) and in serum-free medium (Fig 1B (ii) & (iii)), whereas Nanog and Tbx3 RNA levels were elevated by only ∼ 50% after 8 h, with little or no change in the presence of CHIR (Fig 1A & B (iv)). Furthermore, time-lapse confocal microscopy, using an ESC reporter line in which GFP expression is under the control of the endogenous Nanog promoter (Nanog-TNG [19]), demonstrated that increases in Nanog reporter expression could be detected within 2–4 h of Gsk-3 inhibition (see Fig 1C). Levels of Oct4 protein did not consistently change and while there was a modest increase in c-Myc protein levels in the presence of serum (Fig 1A (iii)), c-Myc RNA levels showed a small decline (Fig 1A (iv)). These data demonstrate that inhibition of Gsk-3 in ESCs leads to relatively selective up-regulation in the levels of Nanog and Tbx3 proteins, which precede changes in RNA levels.

**Figure 2:**
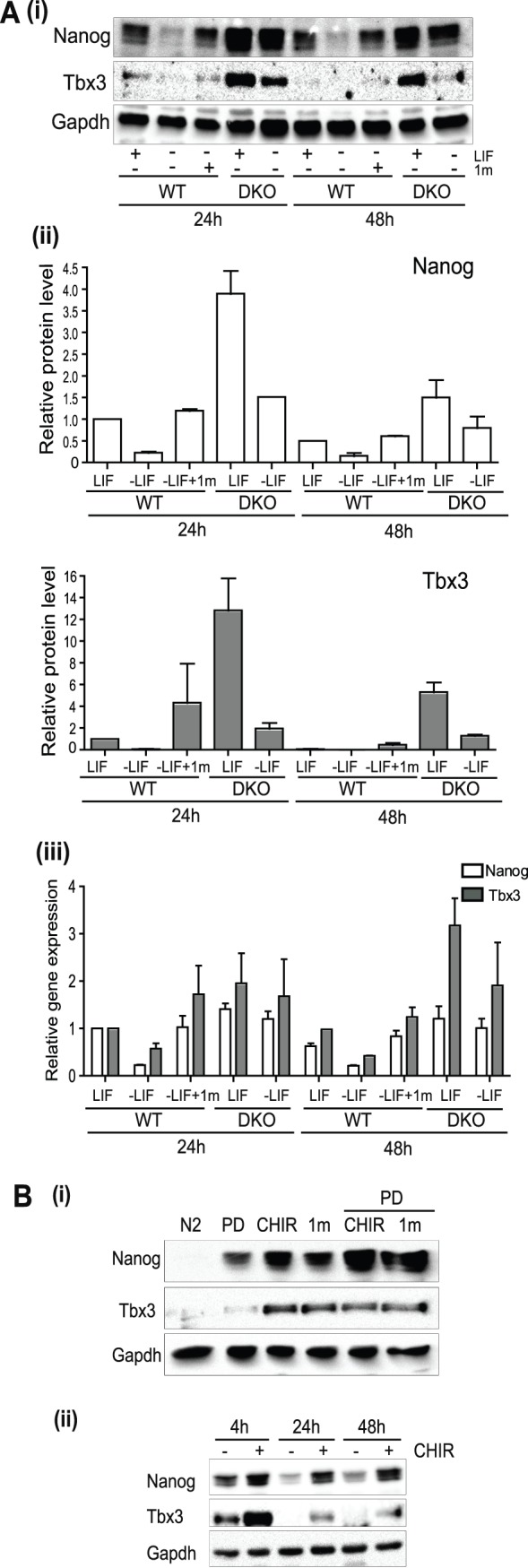
Gsk-3 inhibition or ablation can maintain the expression of pluripotent markers upon LIF withdrawal. (**A**) WT and Gsk-3 DKO ESCs were grown in serum-containing medium in the presence or absence of LIF and WT ESCs were also cultured in the absence of LIF in medium supplemented with 2 µM 1 m. Protein and RNA were extracted at the times indicated. (i) Immunoblotting of 15 µg of nuclear protein was performed with the indicated antibodies. (ii) Antibody signals were quantified and values normalised to Gapdh. A value of 1 was given to normalised protein levels in WT+LIF 24 h sample and values for other samples were related to these. The data show relative protein levels and are the average and SD of duplicate experiments. (iii) Quantitative RT-PCR was carried out and a value of 1 was given to normalised RNA levels in WT ESCs +LIF at 24 h and other samples related to these. The data show relative gene expression levels and are the average and S.E.M of quadruplicate samples. (**B**) (i) E14tg2a ESCs were grown for 24 hours in basal N2B27 medium without LIF or BMP4 in the presence or absence of PD (1 µM), CHIR (3 µM), 1 m (2 µM), CHIR plus PD or 1 m plus PD. Immunoblotting was performed with the antibodies specified. (ii) ESCs were grown for 16 h in basal N2B27 with 1 µM PD before either 3 µM CHIR or vehicle were added for 4, 24 and 48 hrs. Samples were immunoblotted with the antibodies indicated.

We also investigated whether Gsk-3 inhibition influenced expression of Nanog and Tbx3 in the absence of extrinsic factors. In serum-containing medium, Gsk-3 inhibition prolonged expression of Nanog and Tbx3 proteins in the absence of LIF in WT ESC, although after 48 h only low levels of Tbx3 were detected, the reason for which is currently unclear (Fig. 2A(i) and (ii)). Nanog and Tbx3 RNA levels were somewhat down-regulated 24 h after LIF withdrawal in WT cells, an effect reversed by the presence of Gsk-3 inhibitor 1 m (Fig 2A (iii)). In Gsk-3 DKO ESC, although Nanog and Tbx3 RNA levels did not change dramatically upon LIF withdrawal, levels of Nanog and Tbx3 proteins were reduced. The ability of Gsk-3 inhibition to prolong expression of Nanog and Tbx3 in serum-free conditions was also investigated. ESCs were grown in basal N2B27 medium and Gsk-3 (CHIR99201 or 1 m) or MEK (PD) inhibitors were added either alone or in combination to the cultures. After 24 h, Tbx3 and Nanog protein levels were higher in cells treated with Gsk-3 inhibitors compared to cells grown in N2B27 alone or treated with the MEK inhibitor only, while the combination of Gsk-3 and MEK inhibition resulted in maximal levels of Nanog and Tbx3 expression (Fig 2B (i)). ESCs also had a more compact colony morphology after 24 and 48 hours in the presence of Gsk-3 or both inhibitors (see Figure S1). Furthermore, following 16 h culture in N2B27 plus MEK inhibitor alone, addition of CHIR enhanced Nanog and Tbx3 protein levels (Fig 2B(ii)), relative to levels observed in the presence of PD alone. These results provide further evidence to support a role of Gsk-3 in regulation of Nanog and Tbx3 protein expression.

**Figure 3:**
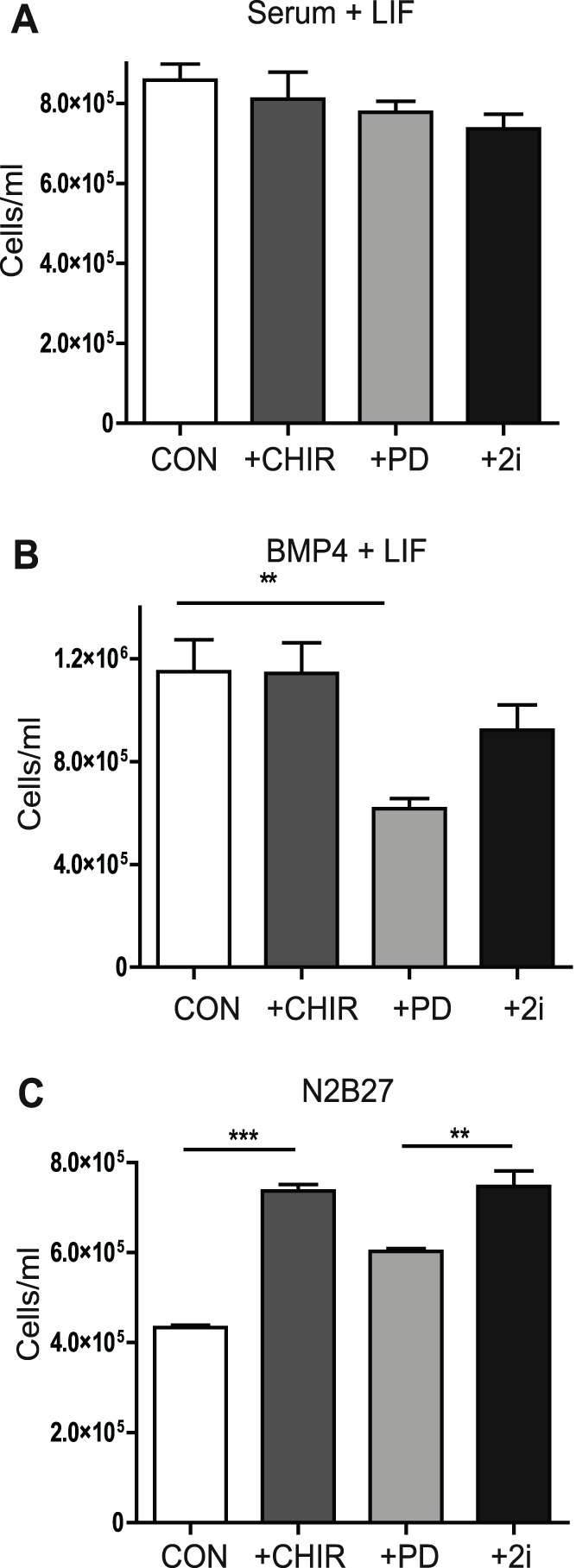
The ability of Gsk-3 inhibition to promote ESC proliferation is dependent upon the culture environment. ESCs were grown in (**A**) GMEM supplemented with Serum and LIF; (**B**) N2B27 supplemented with BMP4 and LIF or **(C**) N2B27 without extrinsic stimuli, in the presence of Gsk3 inhibitor CHIR (CHIR99201) or MEK inhibitor PD (PD0325901) alone or in combination (2i) for 3 days and their growth measured. Control ESCs had DMSO vehicle added instead of inhibitors (CON). The data are the average and S.E.M of triplicate experiments. **, p<0.01, ***, p*<*0.005 (2-way ANOVA with Bonferroni post-hoc test).

It has previously been proposed that inhibition of Gsk-3 enhances the metabolic activity and proliferation of mouse ESCs [15]. Therefore, we investigated whether there was any link between effects on proliferation and enhanced expression of Nanog and Tbx3 proteins, by examining the effects of Gsk-3 and MEK inhibition on ESC growth in the three culture conditions used. In the presence of LIF plus serum no significant effects of CHIR or PD were observed (Fig 3A). In serum-free medium supplemented with LIF and BMP4, inhibition of MEK decreased ESC growth, with proliferation partially restored upon inclusion of the Gsk-3 inhibitor CHIR (Fig 3B). In agreement with previous work [15], in the absence of exogenous factors, Gsk-3 inhibition increased ESC cell growth compared to serum-free medium alone or cultures treated only with PD (Fig 3C). These data suggest that although Gsk-3 inhibition may contribute to maintenance of the ground state of pluripotency by restoring metabolic capacity this is only clearly observed in ground state (2i) conditions [15]. In contrast, under all conditions examined here, inhibition of Gsk-3 resulted in maintenance of protein expression of the pluripotency regulators Nanog and Tbx3.

### Gsk-3 Inhibition does not Alter the Stability of Nanog, Tbx3, Oct4 or c-Myc Proteins

**Figure 4:**
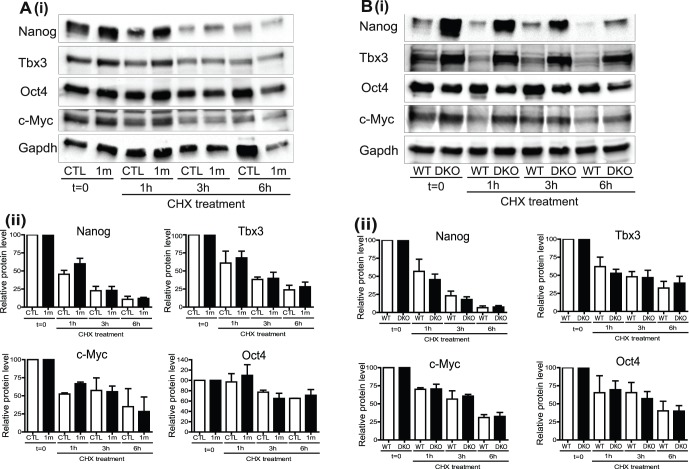
Gsk-3 inhibition does not alter Nanog, Tbx3, Oct4 or c-Myc protein stability. (**A**) E14tg2a WT ESCs and (**B**) WT and Gsk-3 DKO ESCs were grown in the presence of N2B27 plus LIF and BMP4 for 24 h. In (**A**) 1 m (2 µM) or vehicle alone control (CTL) were added to the cells for this 24 h period. All samples were then incubated with cycloheximide (CHX) to halt protein synthesis for the times indicated. (i) Protein samples were extracted after 0, 1, 3 and 6 hours of CHX treatment and immunoblotting performed with the indicated antibodies. (ii) Antibody signals were quantified and protein levels normalised to Gapdh. In each case a value of 100% was given to the t = 0 samples to enable direct comparisons to be made. The data are the average relative protein expression levels and S.E.M of triplicate experiments.

The data presented above raise the possibility that following Gsk-3 inhibition post-transcriptional mechanisms contribute to the increases in protein levels of Nanog, Tbx3 and c-Myc observed. The dynamics of protein expression of these factors has not been previously investigated in ESCs, although Gsk-3 is known to regulate ß-catenin [43] and c-Myc protein stability [7]. Therefore, we examined whether increased protein stability accounted for the increased levels of Nanog, Tbx3 and c-Myc proteins observed following Gsk-3 inhibition. Protein half-lives were estimated by blocking new protein synthesis with cycloheximide and monitoring protein levels over time. In each case, protein levels were normalised relative to Gapdh and levels at t = 0 were set at 100% to enable direct comparison of half-lives in different samples, irrespective of initial relative expression. The results demonstrate that inhibition of Gsk-3 does not appear to consistently affect stability of any of the proteins studied in either N2B27 supplemented with LIF and BMP4 (shown in Figure 4) or in serum plus LIF (see Figure S2). However, that it remains formally possible that in order for Gsk-3 inhibition to extend the protein half-lives of these factors new protein synthesis is required. Interestingly, these data reveal previously undocumented differences in the protein half-lives of these transcription factors. Nanog had the shortest half-life, estimated to be approximately 1 hour; Tbx3 had a half-life of between 1–3 h, c-Myc a half-life of 2–3 h and Oct4 a half-life of 6 h or more. These findings suggest that Nanog, Tbx3 and c-Myc protein levels may be able to change rapidly in response to different stimuli.

### Gsk-3 - a Possible Role in Regulating Translation of Nanog?

Our finding that Nanog and Tbx3 proteins have much shorter half-lives than Oct4 protein raises the possibility that dynamic changes in protein levels allow for rapid responses to environmental signals. However, the stability of Nanog and Tbx3 was not influenced by inhibition of Gsk-3 meaning an alternate mechanism must account for the enhancement in protein levels of these transcription factors observed. General translation has been reported to be low in undifferentiated mouse ESCs and increases as cells differentiate, suggesting that regulation of translation may play a role in controlling stem cell fate [33]. Direct control of protein levels of key transcription factors via translational regulation would represent a new mode by which signalling pathways interact with components of the network of pluripotency transcriptional regulators. Therefore, we decided to investigate a possible role for Gsk-3 in translational regulation of Nanog.

**Figure 5:**
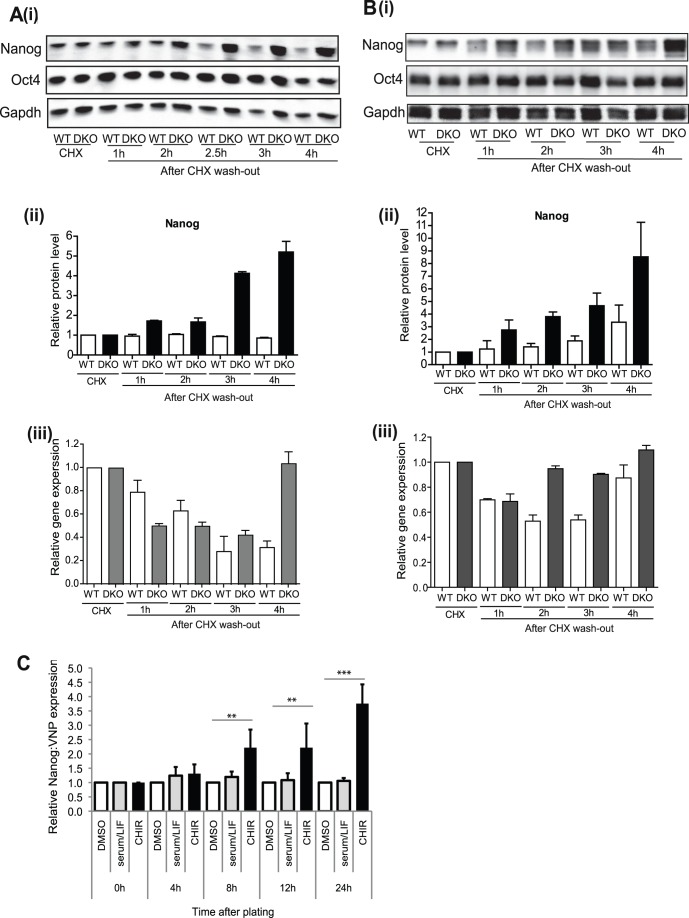
Gsk-3 inhibition or ablation increases Nanog protein synthesis in the absence of increased transcription. WT and Gsk-3 DKO ESCs were incubated in (**A**) GMEM supplemented with LIF and serum or (**B**) N2B27 medium supplemented with LIF and BMP4 prior to addition of 10 mg/ml CHX for 4 hours to halt protein synthesis. CHX was then washed out and fresh media (**A**, GMEM plus LIF plus serum and **B**, N2B27 plus LIF and BMP4) added back. (i) & (ii) Protein and (iii) RNA were extracted between 1 and 4 hours after CHX washout. (i) Immunoblotting was performed with the antibodies indicated. (ii) Antibody signals were quantified and normalised to Gapdh. A value of 1 was given to CHX-treated samples. The data are the average relative protein expression levels and SD of duplicate experiments. (iii) Quantitative RT-PCR was carried out and Nanog expression normalized relative to ß-actin levels. A value of 1 was given to CHX-treated samples in (iii). The data are the average gene expression levels and S.E.M of quadruplicate samples. (**C**) Nanog reporter Nd ESCs were sorted by flow cytometry and cells with low to no VNP expression collected and grown in GMEM supplemented with LIF and serum in the presence or absence of DMSO (controls) or in the presence of 3 µM CHIR99201. The data shown are the average Nanog:VNP expression levels (relative to DMSO control) and standard deviation of at least 3 biological replicates. All *p*-values were calculated using a two-tailed distribution, two-sample equal variance t-test (** p-value <0.01; *** p-value <0.0001).

To examine effects of Gsk-3 on *de novo* protein synthesis of Nanog, we performed a series of protein re-synthesis experiments. Protein synthesis was halted by addition of cycloheximide and re-synthesis initiated by its removal, extensive washing and addition of fresh medium. Increases in Nanog protein levels were observed as early as 1–2 hours after CHX washout in the Gsk-3 DKO ESCs, whereas in WT ESCs, Nanog protein recovery was not observed even after 4 hours (Fig 5A (i) and (ii)). Nanog RNA levels in the same samples did not significantly increase even 3 h after CHX washout, compared to CHX-treated (Fig 5A (iii)). Results demonstrating a similar trend were obtained when this experiment was conducted in serum-free N2B27 medium in the presence of BMP4 and LIF (Fig. 5B). In no situation were changes in levels of Oct4 observed and our results are consistent with Nanog protein re-synthesis in Gsk-3 DKO ESCs occurring without corresponding increases in Nanog RNA levels. Treatment of WT ESCs with Gsk-3 inhibitor 1 m also led to enhanced re-synthesis of Nanog protein (shown in Figure S3). In these latter analyses we noted that ß-catenin levels do not decline significantly following 4 h treatment with cycloheximide, whereas Nanog levels do. In contrast, after a 16 h recovery phase in the absence of Gsk-3 inhibition, Nanog levels have approximately doubled, while ß-catenin levels have approximately halved (compared to levels after cycloheximide treatment). These data demonstrate a disconnection between Nanog and ß-catenin levels.

Next, we sought to confirm these data using an approach that did not rely on the use of cycloheximide. To achieve this we used an mESC reporter line in which the coding sequence for the fluorescent protein VNP (Venus) had been inserted into the endogenous Nanog locus (Nd-ESCs; [30]). In this cell line, VNP expression is under the control of endogenous sequences that influence Nanog expression. Furthermore, VNP has a half-life of 2–3 h, which is very similar that of Nanog, making this an ideal reporter for our studies. Flow cytometry was used to sort a population of Nanog:VNP cells with low levels of VNP (Nanog) expression (see Figure S4). These cells were immediately plated into media containing serum and LIF, in the presence or absence of Gsk-3 inhibitor, and the reappearance of VNP expression monitored over a period of 24 h. Images of the replated cells are shown in Figure S4. As shown in Figure 5C, the proportion of VNP positive cells is increased in the presence of Gsk-3 inhibitor, when compared to control cells, while cell viability was maintained at over 95% in each condition. The increase in the proportion of Nanog:VNP positive cells was significant from 8 h following re-plating of the Nanog:VNP-low population, consistent with Gsk-3 inhibition enhancing protein resynthesis. Thus, based on two alternate approaches, our results are consistent with Gsk-3 playing a role in regulating translation of Nanog.

### Gsk-3 Inhibition Increases Association of Nanog RNA with Polyribosomes

**Figure 6:**
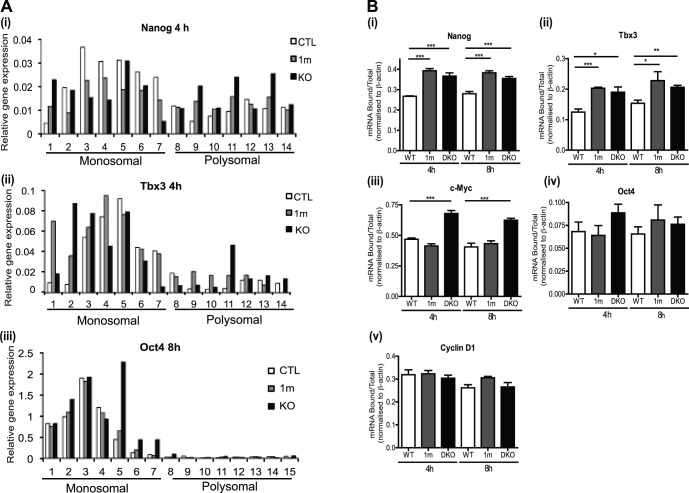
Association of Nanog and Tbx3 RNAs with polysomes increases upon inhibition of Gsk3. WT ESCs grown in the absence (WT) or presence of 2 µM 1 m (1 m) and Gsk-3 DKO ESCs (DKO) were cultured in serum supplemented with LIF for 4 and 8 hours before extracting cell lysates. Sedimentation through sucrose gradients was used to separate the polysomal-enriched fractions from the monosomal fractions. The levels of RNA bound to polysomal or monosomal fractions were measured using quantitative RT-PCR, with the primers shown in Table S1. In each case gene expression was normalized relative to ß-actin levels. **A.** Expression of (i) Nanog, (ii) Tbx3 and (iii) Oct4 in individual fractions across the sedimentation gradient. A representative experiment is shown in each case (4 h time points for (i) and (ii) and 8 h time point for (iii)). Values show expression relative to ß-actin. **B.** Gene expression in pooled monosomal and polysomal fractions. Values show the proportion of RNA bound to the polysome fraction (Bound/Total RNA). The data are the average and S.E.M of three independent experiments run in duplicate for the 8-hour time point and the average and S.E.M of two independent experiments run in duplicate for the 4-hour time point. *, p<0.05; **, p<0.01, ***, p<0.005 (Student’s t-test).

Our data demonstrate that the increase in Nanog protein observed between 1 and 3 h in Gsk-3 DKO ESCs following release of CHX block does not directly correlate with increases in Nanog RNA. However, at later time points, for example 24 h following addition of 1 m, we have observed modest increases in Nanog RNA (Fig. 1A,B (iii)). These findings suggest that while Gsk-3 may contribute to regulation of Nanog transcription at later time points, at early stages following Gsk-3 inhibition, Gsk-3′s main role may be in regulating Nanog translation. To further investigate a possible role for Gsk-3 in regulating translation of Nanog, and other pluripotency-associated transcription factors, we examined association of their RNAs with polyribosomes. RNAs associated with polyribosomes are more actively translated hence a shift in the proportion of RNA encoding a particular protein to the polysomal fraction is consistent with enhanced translation of that protein [44]. Monosomal and polysomal-enriched fractions of RNA were generated by differential sucrose density centrifugation. We analysed the distribution of Nanog, Tbx3 and Oct 4 RNAs across the entire gradient (see Figure 6A) and we also pooled fractions into generate monosomal and polysomal samples, allowing us to perform statistical analyses (see Figure 6B). As can be seen in Figure 6A, inhibition of Gsk-3 leads to an increase in Nanog RNA associated with polysomes, with two populations of Nanog RNAs clearly detected. In contrast, only very low levels of Oct4 RNA were detected in the polysomal fractions and these did not appear to alter upon inhibition of Gsk-3. Tbx3 RNAs also showed an increase in the polysomal fractions following inhibition of Gsk-3. When we analysed the distribution within the pooled monosomal and polysomal fractions, increases of between 30 and 45% in the proportion of Nanog and Tbx3 RNAs bound to polysomes were observed after 4 and 8 h treatment of WT ESCs with the Gsk-3 inhibitor 1 m and in Gsk-3 DKO ESCs and these were statistically significant (Figure 6B). On the other hand, no significant differences were observed in the distribution of Oct4 and CyclinD1 RNAs between polysomal and monosomal fractions. In the case of c-Myc a significant difference was only observed in Gsk-3 DKO ESCs, suggesting sustained inhibition of Gsk-3 is needed to increase loading of c-Myc RNA to polysomes. The increase in the proportion of Nanog RNA associated with the polyribosomes in 1m-treated and Gsk-3 DKO ESC is consistent with an increase in Nanog translation following inhibition of Gsk-3 activity and correlates with enhancement in *de novo* protein synthesis.

### Inhibition of Gsk-3 in Mouse ESCs does not Affect Regulators of General Translation Initiation Machinery

**Figure 7:**
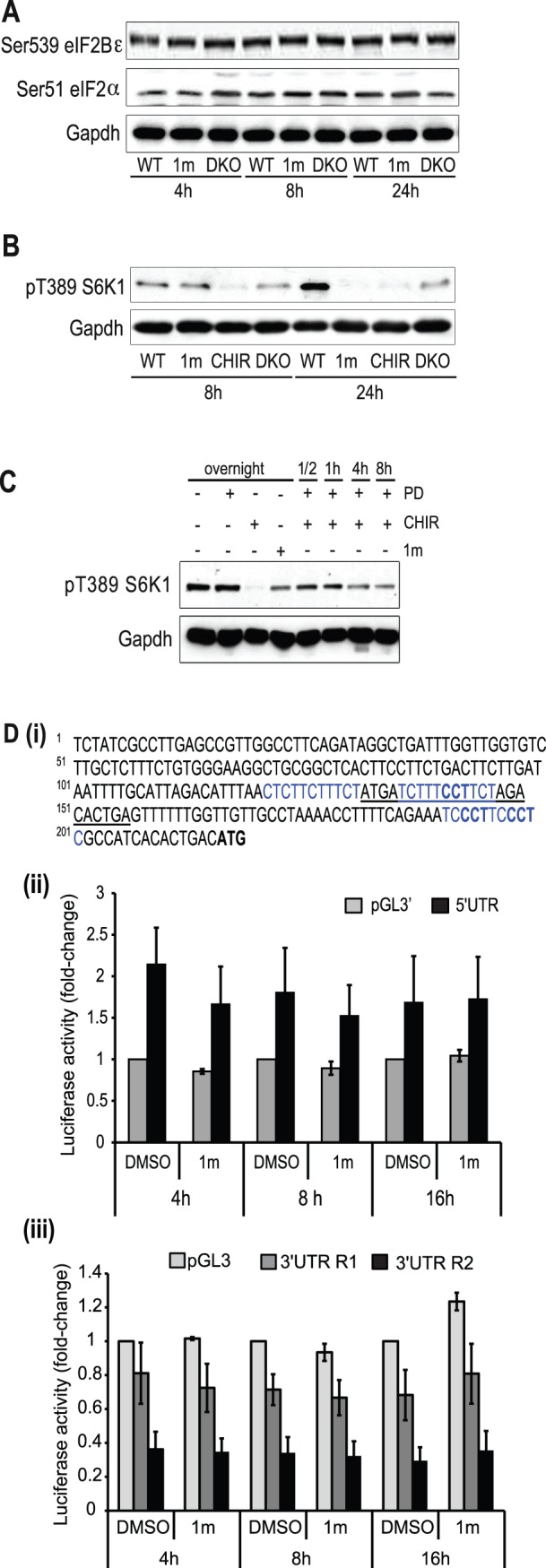
Gsk-3 does not mediate its effects via general translational machinery or the 5′UTR of Nanog. (**A**) E14tg2a wild-type (WT) and Gsk-3α/ß double knockout (DKO) ESCs were cultured in the presence of LIF and serum. Gsk-3 inhibitor 1 m was added to WT cells at 2 µM and protein samples taken at the times indicated. Cell lysates were blotted with the antibodies indicated. (**B**) WT and Gsk-3 DKO ESCs were grown in N2B27 plus BMP4 and LIF. WT were treated with 2 µM 1 m or 3 µM CHIR for 8 and 24 hours before lysis. Immunoblotting was performed with antibodies against pT389 S6K1 and Gapdh. (**C**) E14tg2a ESCs were grown overnight in the presence of MEK inhibitor (PD) before being treated with 3 µM CHIR or 2 µM 1 m for the times indicated. ESCs were also grown in either CHIR or 1 m alone overnight prior to preparation of cell lysates. Immunoblotting to detect phosphorylation of T389 on S6K1 and Gapdh were performed. (**D**)(**i**) The 215bp 5′UTR sequence of the mouse Nanog gene (RefSeq NM_028016). Polypyrimidine tracts, shown in blue, were identified at bp positions 122, 137 and 191, with the tract at 137 containing 1 CCT motif and the tract at position 191 containing 2 CCT motifs (in bold font). One potential uORF, underlined, with an ATG start codon was identified between bp133 to 156. WT E14 ESCs were transfected with (**ii**) pGL3’ or pGL3’-Nanog-5′UTR in the presence of pRenilla luciferase plasmid or (**iii)** pGL3-MCS, pGL3-Nanog-3′UTR R1 or pGL3-Nanog-3′UTR R2 in the presence of pRenilla luciferase plasmid. After 24 h, transfected cells were treated with vehicle alone (C) or 2 µM 1 m. Cells were harvested at 4, 8 and 16 h and Firefly luciferase activity determined and normalised relative to Renilla luciferase activity. Luciferase activity is expressed as fold-change over pGL3’ (**ii**) or pGL3-MCS (**iii**) controls (DMSO) for a given time point. Data are the average and S.E.M. of three independent experiments.

Next, we were interested to investigate the mechanism mediating the effects of Gsk-3 inhibition on Nanog translation. A previous report has suggested that general translation rates are increased as mouse ESCs differentiate [33], indicating that translational control may be involved in regulation of ESC fate and self-renewal. Our studies have demonstrated that Gsk-3 inhibition increases the expression of pluripotency-associated transcription factors Nanog and Tbx3 and that their RNAs appear to be more actively translated. Therefore, we investigated the effect that Gsk-3 inhibition has on key regulators of translation initiation in somatic cells. The activity of the initiation factor eukaryotic translation factor 2 (eIF2) and its guanine nucleotide exchange factor, eIF2Bε, are regulated by phosphorylation. Gsk-3, downstream of PI3K, phosphorylates Ser 539 of eIF2Bε resulting in its inactivation [45], [46], while phosphorylation of Ser51 on eIF2α prevents binding of eIF2Bε to eIF2α. Thus, decreased phosphorylation of both sites would be consistent with increased translation initiation of most mRNAs [47], [48]. We examined the levels of phosphorylation of Ser539 on eIF2Bε and Ser51 on eIF2α in WT ESCs grown in the presence and absence of 1m and in Gsk-3 DKO ESCs. Phosphorylation of neither of these regulatory sites was altered following perturbation of Gsk-3 activity (Fig 7A), suggesting general translation initiation events are not significantly affected, consistent with our polysomal data where only a subset of pluripotency factors are targeted by Gsk-3, e.g. Nanog and Tbx3.

Another pathway that regulates protein synthesis is the mTor pathway. Gsk-3 can inhibit the mTor pathway by phosphorylating TSC2 at Ser1337 and Ser1341 leading to TSC2 activation and the subsequent inhibition of Rheb and mTor activity [49]. A key downstream effector of mTor is S6K1, which can regulate translation initiation, elongation and ribosome biogenesis and was, therefore, of particular interest. Surprisingly, we discovered that levels of S6K1 T389 phosphorylation, the site phosphorylated by mTor, were reduced following treatment of ESCs with 1 m or CHIR and in Gsk-3 DKO ESCs (Fig 7B). This effect appears selective as 1 m had no effect on T389 phosphorylation in Gsk-3 DKO ESCs (see Figure S5). We also studied changes in T389 phosphorylation in cells grown with CHIR or 1 m overnight in comparison with PD alone (Fig 7C). Consistent with the results shown in Fig 7B, in the presence of Gsk-3 inhibitors, levels of phosphorylation of S6K1 at T389 were reduced. Furthermore, when CHIR was added to cells that had been treated for 16 h with PD alone, phosphorylation of T389 declined rapidly. Taken together, these results suggest that inhibition of Gsk-3 in ESCs does not lead to general increases in translation. This supports our polysomal distribution data, where proportions of Oct4 and CyclinD1 are unaffected, arguing against a general enhancement in translation and in favour of selective action of Gsk-3 in controlling protein levels of a subset of factors involved in regulation of pluripotency.

Given that Gsk-3 inhibition does not appear to be acting via regulation of the general translation initiation machinery, we were interested to examine whether the 5′ or 3′ untranslated regions of Nanog are responsible for mediating the effects of Gsk-3 inhibition. Our bioinformatic analyses demonstrate that the 215 base pair 5′UTR region of the mouse Nanog gene contains three polypyrimidine tracts, which represent potential internal ribosomal entry sites, and one potential upstream open reading frame (uORF) with an ATG start codon, shown in Figure 7D(i). A number of uORFs with non-ATG start codons were also identified (spanning bases 10–99, 18–32, 39–113, 62–91, 153–176 and 166–219). The 1048 bp 3′ UTR region of the mouse Nanog gene examined contained only weakly conserved ARE sites and poorly conserved miRNA binding sites (not shown). To determine whether the Nanog 5′UTR sequence mediates the effects of Gsk-3 inhibition on Nanog translation, we cloned this 215 bp sequence into the pGL3’ vector [35] so that it was upstream of the SV40-promoter driving Firefly luciferase. pGL3’ and pGL3’-Nanog-5′UTR were co-transfected with a Renilla-encoding plasmid into E14 ESCs and 24 h following transfection, 1 m added for 4, 8 or 16 h. Our results demonstrate that the 5′UTR region of Nanog enhances expression of luciferase by approximately 2-fold but that treatment with 1 m has no additional effect (shown in Fig 7D(ii)). In view of this, next we examined whether 222 bp or 1048 bp segments of the Nanog 3′ UTR region played a role in regulation of Nanog translation in response to Gsk-3 inhibition. As shown in Fig 7D (iii), inclusion of 222 bp of Nanog 3′ UTR sequence led to a 20–30% decrease in luciferase activity, with the 1048 bp segment of the Nanog 3′UTR reducing luciferase activity by over 60%. Despite these changes, Gsk-3 inhibition had no significant effect on luciferase activity levels. These results suggest that in isolation, neither the 5′UTR nor 3′UTR regions of Nanog mediate the effects of Gsk-3 inhibition on Nanog translation.

## Discussion

The data we present here provide new insight into the mechanisms regulated by Gsk-3 in ESCs. We show that inhibition or ablation of Gsk-3 activity enhances the protein levels of the pluripotency-associated transcription factors Nanog, Tbx3 and c-Myc. The stability of these proteins were not altered, but rather we demonstrate, using two approaches, that the *de novo* synthesis of Nanog protein is increased following Gsk-3 inhibition. We also report corresponding increases in polyribosome association of Nanog and Tbx3 RNAs following Gsk-3 inhibition, consistent with enhanced translation. Based on our data, we propose that Gsk-3 is involved in the regulation of dynamically expressed pluripotency-associated factors in mouse ESCs, including Nanog and Tbx3, by altering their translation. This represents a novel additional mechanism by which Gsk-3 may contribute to modulation of ESC self-renewal and highlights the role that control of translation can play in fine-tuning protein levels to reinforce the network of pluripotency factors. Furthermore, our study provides additional evidence regarding the coupling of molecular signals to transcriptional control in ESCs.

A number of possible mechanisms have been proposed to account for the ability of Gsk-3 inhibition to enhance self-renewal of mouse ESCs, including promotion of ESC survival and metabolism [15], stabilisation of c-Myc [42], [50] and activation of Wnt signalling [13], [50], [51], [52], [53], [54]. This latter aspect had proven to be somewhat controversial, but several recent reports suggest that inhibition of Gsk-3 leads to enhancement of ß-catenin levels, which binds to Tcf3 thereby relieving the repressive effects of Tcf3 on expression of genes of the pluripotency network [38], [39], including Esrrb [27]. Our study suggests an alternative additional mechanism that may also contribute to modulation of self-renewal and reveals how the signals known to impact on control of pluripotency can influence the transcriptional machinery. While Gsk-3 inhibition did not, in our analyses, appear to alter the stability of Nanog, Tbx3, c-Myc or Oct-4 proteins, our results reveal an interesting difference in the half-lives of Nanog and Tbx3 proteins (between 1 and 3 hours) compared to Oct4, which had a half-life in the order of 6-plus hours. These data suggest that owing to their dynamic turnover, Nanog and Tbx3 are potentially more responsive to the signalling environment and any changes that may occur. Thus, by enhancing translation of Nanog and Tbx3, inhibition of Gsk-3 may act to reinforce the network of pluripotency-associated transcription factors in a dynamic and responsive manner. While the differences in polysomal-bound Nanog mRNA following Gsk-3 inhibition may not appear to increase to the same degree as Nanog protein levels observed in Figure 1, it is likely that each polysome-associated Nanog mRNA is translated numerous times, which would increase total protein output compared to the underlying proportion of polysome-bound RNA.

Previous reports have suggested that Nanog binds to its own promoter, enhancing its transcription [23], [55], although in contrast a very recent report suggests that Nanog is auto-repressive [56]. How then, do our data relate to these somewhat conflicting findings? We propose that the increased levels of Nanog protein, caused by increased translation as a result of Gsk-3 inhibition, could contribute to the fine-tuning of Nanog protein levels in response to the external environment, providing a means for more rapid and dynamic regulation of pluripotency factors than allowed for by transcriptional changes alone, a concept not previously considered [57]. While the focus of this study has been on the early events arising as a result of Gsk-3 inhibition, this mechanism could contribute to sustaining ESC pluripotency whenever Gsk-3 inhibition is invoked. Indeed, there are many examples where short-term signalling events, e.g. receptor phosphorylation, have significant effects on cellular responses occurring hours later.

In our study we were interested to determine the mechanism underlying the ability of Gsk-3 to modulate protein translation. Interestingly, when we investigated whether inhibition of Gsk-3 in ESCs led to a change in regulators controlling general (CAP-dependent) initiation of translation, no alteration in phosphorylation of either Ser51 on eIF2α or Ser 539 on eIF2Bε phosphorylation were observed. These results suggest that general translation initiation is not perturbed following Gsk-3 inhibition in ESCs, consistent with the fact that only a subset of pluripotency factors are targeted by Gsk-3, e.g., Nanog and Tbx3. These results are interesting in view of the fact that general translation has been reported to be lower in undifferentiated ESCs, increasing following differentiation into embryoid bodies [33]. We also discovered that mTor activity did not appear to increase following Gsk-3 inhibition and unexpectedly phosphorylation of T389 on S6K1 declined. The effect of Gsk-3 inhibition on levels of T389 S6K1 phosphorylation were consistently observed following treatment with structurally unrelated Gsk-3 inhibitors (1 m and CHIR) and also evident in Gsk-3 null ESCs. One possible explanation for this observation is that it has recently been reported that Gsk-3-phosphorylates S371 of S6K1 [58]. S371 phosphorylation of S6K1 is essential for phosphorylation of T389 [59] so it is possible that the decrease in T389 phosphorylation we observe is due to inhibition of Gsk-3-mediated phosphorylation of S371. Further studies are required to examine this in detail and determine any functional significance in mouse ESCs.

The fact that translation of other genes, including Oct4 and Cyclin D1, are not increased following Gsk-3 inhibition and the lack of effect on regulators of CAP-dependent initiation of translation suggests that the increase in translation observed with Nanog and Tbx3 are, at least partially, selective. Translation of specific RNA transcripts, without an increase in general translation or in conditions where the CAP-dependent translation is compromised, can occur via a number of different mechanisms including IRES-based translation, control by miRNAs or via RNA binding protein-mediated mechanisms. Our bioinformatic studies predicted that the 5′UTR of Nanog contains three possible IRES and a potential in-frame upstream open reading frame, so we were interested to investigate whether it mediated the effects of Gsk-3 inhibition observed. Our functional analyses show that while the Nanog 5′UTR enhances reporter gene expression, by itself it does not confer responsive to Gsk-3 inhibition. Our further analyses demonstrate a lack of conservation in the Nanog 5′UTR sequences across a range of species. We also discovered that Nanog 3′UTR sequences do not appear to independently mediate the effects of Gsk-3 inhibition, thus, it may be that a combination of 5′ and 3′ UTR sequences are required. Another alternate possibility is that ß-catenin mediates the effects of Gsk-3 inhibition, given its reported role in maintaining ESC self-renewal [37], [38], [50], [52], [60], [61]. Interestingly, while the levels of nuclear ß-catenin reflect the enhanced re-synthesis of Nanog following 1 m treatment (Figure S3), the changes in the levels of Nanog and ß-catenin protein do not correlate well following either short-term cycloheximide treatment or a 16 h recover phase in the absence of Gsk-3 inhibition, suggesting the effects on Nanog protein levels are at least partially ß-catenin-independent. In future studies it will be interesting to examine the ß-catenin-independence/dependence in greater detail.

In this study we present evidence supporting a role for Gsk-3-dependent regulation of translation of selected pluripotency-associated transcription factors that show dynamic protein turnover. We propose that this represents a novel additional mechanism that may contribute to modulation of ESC pluripotency by enabling rapid responses to changes in signals, leading to alteration of protein levels that act to reinforce the network of pluripotency-associated transcription factors. This study highlights the importance of interrogating the control of protein dynamics and translational regulation to gain further insight into the mechanisms contributing to the control of ESC fate.

## Supporting Information

Figure S1 **Colony morphology of E14 mouse ESCs cultured in N2B27 medium in the different conditions indicated.**
(TIF)

Figure S2 **Gsk-3 inhibition does not alter Nanog, Tbx3, Oct4 or c-Myc protein stability in the presence of serum and LIF.** ESCs were grown in GMEM plus serum plus LIF. (A) E14tg2a WT ESCs were pre-incubated for 24 h without (CTL) or with 2 µM 1 m. (B) WT and Gsk-3 DKO ESCs were cultured for 24 h before addition of cycloheximide (CHX) to halt protein synthesis. (i) Protein samples were extracted after 0, 1, 3 and 6 hours of CHX treatment and immunoblotting performed with the indicated antibodies. (ii) Antibody signals were quantified and protein levels normalised to Gapdh. In each case a value of 100% was given to the untreated (t = 0) samples to enable direct comparisons to be made. The data are the average and S.E.M of triplicate experiments.
(EPS)

Figure S3 **Gsk-3 inhibition increases Nanog protein synthesis.** E14tg2a mouse ESCs were incubated with cycloheximide (CHX) for 4 h to halt protein synthesis or left untreated (UT) as a control. Cells were washed thoroughly to remove CHX and fresh media supplemented with serum and LIF and vehicle (DMSO/D) or 2 µM 1 m (1 m) added back. Cells were harvested and protein extracts prepared at the times indicated after CHX wash-out. (ii) Immunoblotting was performed with the antibodies indicated. (iii) Nanog and ß-catenin expression levels normailsed to Gapdh and expressed relative to the UT control are shown following the 16 hr treatment. The values are the average and S.E.M. from 3 independent experiments.
(TIF)

Figure S4 **Gsk-3 inhibition increases Nanog protein synthesis in Nd ESCs.** Nd ESCs were sorted and the VNP-low sub-population collected and incubated in GMEM supplemented with LIF and serum in the presence or absence of DMSO (controls) or in the presence of 3 µM CHIR99201. (**A**) Representative bright field images of Nd ESCs at 4 h and 24 h after plating. Scale-bar = 100 µm. (**B**) Representative dot blots for Nanog:VNP expression at 4 h and 24 h after plating. VNP-low FACS sorted populations of Nd ESCs were plated in the conditions indicated and reporter expression measured after 4 and 24 h. At 4 h no differences were observed, while at 24 h a statistically significant increase in the number of Nanog:VNP positive cells was observed. (**C**) Relative Nanog:VNP expression after plating of low-Nanog sorted population. In the presence of CHIR99201, Nanog:VNP expression increases approximately 30-fold relative to time 0 h, while in DMSO only a 10-fold increase is observed. No differences were observed between GMEM supplemented with LIF and serum in the presence or absence of DMSO. The data are the average relative Nanog:VNP expression levels (relative to time 0 h) and Standard Deviations of at least 3 biological replicates. E14tg2a cells were used as a negative control, to obtain the positive gate region. All *p*-values were calculated using a two-tailed distribution, two-sample equal variance t-test. For 8, 12 and 24 h time points p<0.05.
(TIFF)

Figure S5 **Gsk-3 inhibitors do not alter T389 phosphorylation of S6K1 in Gsk-3 DKO ESCs.** Gsk-3 DKO ESCs were cultured in serum plus LIF for 24 h before LIF was removed from the cells for 4 h. Cells were then pre-treated for 30 mins with 5 µM LY294002 (PI3k inhibitor), 10 nM Rapamycin (mTor inhibitor) or 2 µM 1 m (GSk-3 inhibitor). Cells were then treated for 10 mins with 1000 U/ml LIF prior to generation of cell extracts. Immunoblotting was performed with the antibodies indicated.
(EPS)

Table S1 **Sequences of primers used in this study for quantitative PCR.**
(DOC)

Video S1 Nanog-TNG (GFP-reporter) ESCs were plated in GMEM supplemented with Serum and LIF overnight and then equilibrated for 1 h on the confocal microscope at 37°C, prior to addtion of DMSO. Time-lapse was conducted for a period of 24 h, with images taken every 10 mins. The video shows 10 frames per second.
(AVI)

Video S2 Nanog-TNG (GFP-reporter) ESCs were plated in GMEM supplemented with Serum and LIF overnight and then equilibrated for 1 h on the confocal microscope at 37°C, prior to addtion of 3 µM CHIR99201 (corresponding to CHIR-2 in Figure 1). Time-lapse was conducted for a period of 24 h, with images taken every 10 mins. The video shows 10 frames per second.
(AVI)
