# Supplementary figures and images for: Glycogen Synthase Kinase-3 Inhibition Enhances Translation of Pluripotency-Associated Transcription Factors to Contribute to Maintenance of Mouse Embryonic Stem Cell Self-Renewal
Source: PLoS One. 2013 Apr 5;8(4):e60148. doi: 10.1371/journal.pone.0060148 (PMC3618116; doi:10.1371/journal.pone.0060148)

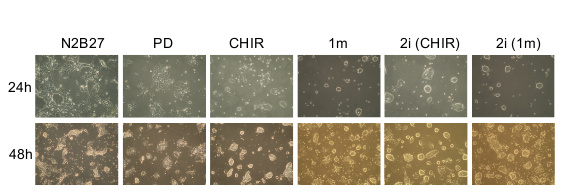

Supplement: Figure S1 — Colony morphology of E14 mouse ESCs cultured in N2B27 medium in the different conditions indicated. (TIF) [file pone.0060148.s001.tif]

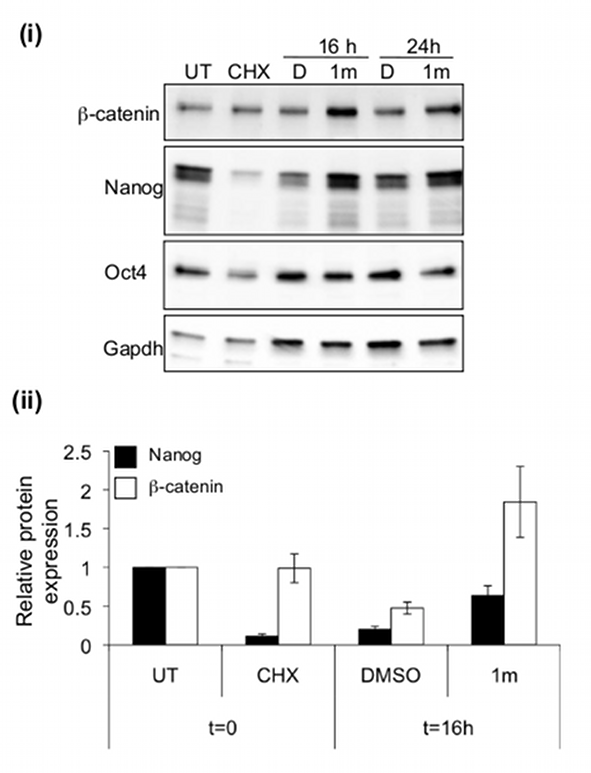

Supplement: Figure S3 — Gsk-3 inhibition increases Nanog protein synthesis. E14tg2a mouse ESCs were incubated with cycloheximide (CHX) for 4 h to halt protein synthesis or left untreated (UT) as a control. Cells were washed thoroughly to remove CHX and fresh media supplemented with serum and LIF and vehicle (DMSO/D) or 2 µM 1 m (1 m) added back. Cells were harvested and protein extracts prepared at the times indicated after CHX wash-out. (ii) Immunoblotting was performed with the antibodies indicated. (iii) Nanog and ß-catenin expression levels normailsed to Gapdh and expressed relative to the UT control are shown following the 16 hr treatment. The values are the average and S.E.M. from 3 independent experiments. (TIF) [file pone.0060148.s003.tif]

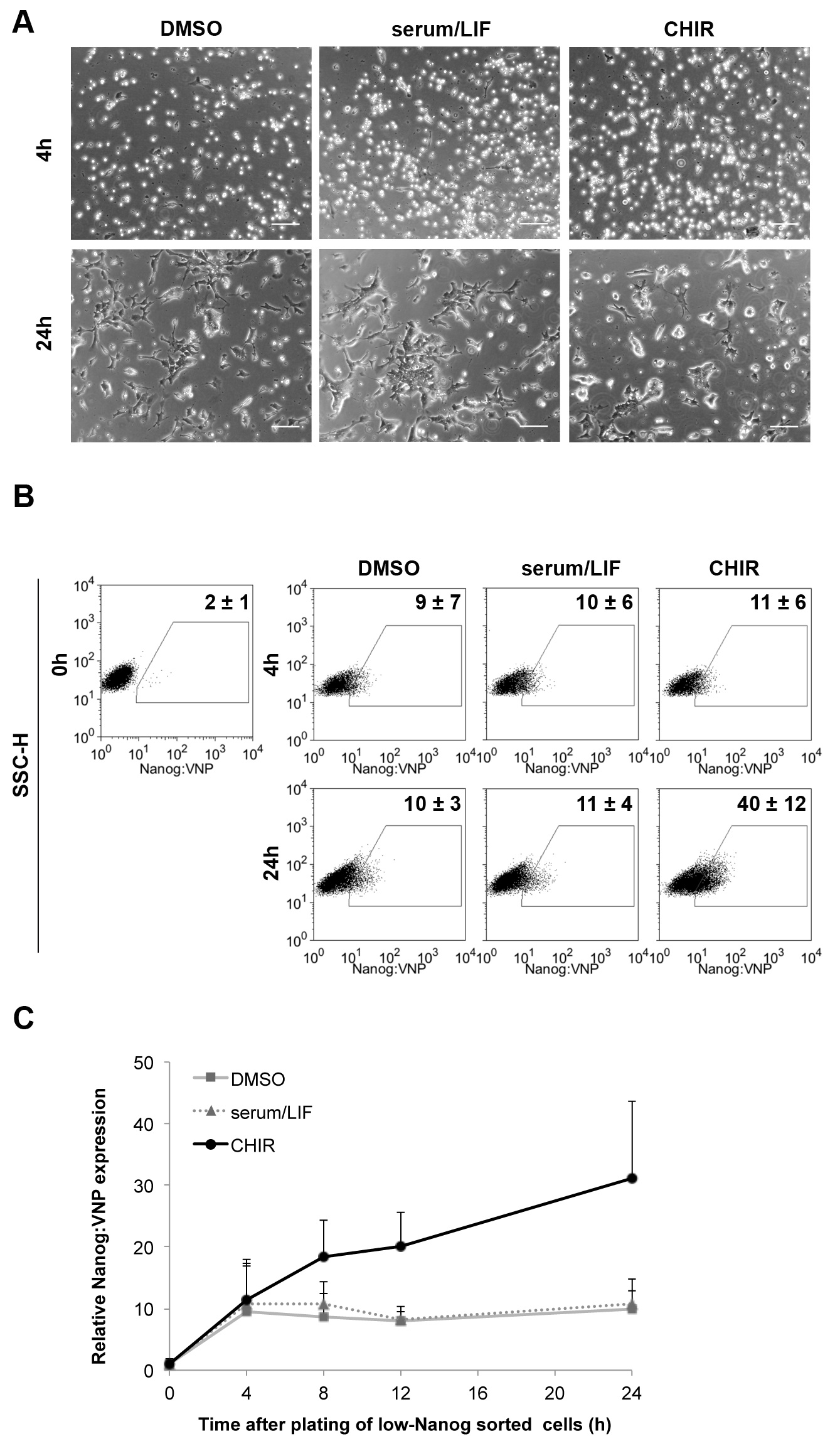

Supplement: Figure S4 — Gsk-3 inhibition increases Nanog protein synthesis in Nd ESCs. Nd ESCs were sorted and the VNP-low sub-population collected and incubated in GMEM supplemented with LIF and serum in the presence or absence of DMSO (controls) or in the presence of 3 µM CHIR99201. (A) Representative bright field images of Nd ESCs at 4 h and 24 h after plating. Scale-bar = 100 µm. (B) Representative dot blots for Nanog:VNP expression at 4 h and 24 h after plating. VNP-low FACS sorted populations of Nd ESCs were plated in the conditions indicated and reporter expression measured after 4 and 24 h. At 4 h no differences were observed, while at 24 h a statistically significant increase in the number of Nanog:VNP positive cells was observed. (C) Relative Nanog:VNP expression after plating of low-Nanog sorted population. In the presence of CHIR99201, Nanog:VNP expression increases approximately 30-fold relative to time 0 h, while in DMSO only a 10-fold increase is observed. No differences were observed between GMEM supplemented with LIF and serum in the presence or absence of DMSO. The data are the average relative Nanog:VNP expression levels (relative to time 0 h) and Standard Deviations of at least 3 biological replicates. E14tg2a cells were used as a negative control, to obtain the positive gate region. All p-values were calculated using a two-tailed distribution, two-sample equal variance t-test. For 8, 12 and 24 h time points p<0.05. (TIFF) [file pone.0060148.s004.tif]
