# Supplementary material for: Glycogen Synthase Kinase-3 Inhibition Enhances Translation of Pluripotency-Associated Transcription Factors to Contribute to Maintenance of Mouse Embryonic Stem Cell Self-Renewal
Source: PLoS One. 2013 Apr 5;8(4):e60148. doi: 10.1371/journal.pone.0060148 (PMC3618116; doi:10.1371/journal.pone.0060148)
Supplement: Table S1 — Sequences of primers used in this study for quantitative PCR. (DOC) [file pone.0060148.s006.doc]

| **Gene** | **Primer Sequences** | | **Annealing**  **Temperature** |
| --- | --- | --- | --- |
| β-actin | sense | 5'-TAGGCACCAGGGTGTGATGG | 62ºC |
|  | anti-sense | 5'-CATGGCTGGGGTGTTGAAGG |  |
| Nanog | sense | 5'-CTCTTCAAGGCAGCCCTGAT | 60ºC |
|  | anti-sense | 5'-CCATTGCTAGTCTTCAACCAC |  |
| Oct4 | sense | 5'GGCGTTCTCTTTGGAAAGGTGTTC | 58ºC |
|  | anti-sense | 5'-CTCGAACCACATCCTTCTCT |  |
| Tbx3 | sense | 5´-AGGAGCGTGTCTGTCAGGTT | 58ºC |
|  | anti-sense | 5´-GCCATTACCTCCCCAATTTT |  |
| c-Myc | sense | 5’CAGAGGAGGAACGAGCTGAAGCGC | 58ºC |
|  | anti-sense | 5´-GGCTTCAATCTGTTCCTGGC |  |
| CyclinD1 | sense | 5´-CTACACTGACAACTCTATCC | 56ºC |
|  | anti-sense | 5´-GGCTTCAATCTGTTCCTGGC |  |

**Table S1. Sequences of primers used in this study for quantitative PCR**
